# Sputtering Codeposition and Metal-Induced Crystallization to Enhance the Power Factor of Nanocrystalline Silicon

**DOI:** 10.1021/acsaelm.2c01772

**Published:** 2023-04-04

**Authors:** Andres Conca, Elías Ferreiro-Vila, Alfonso Cebollada, Marisol Martin-Gonzalez

**Affiliations:** Instituto de Micro y Nanotecnología, IMN-CNM, CSIC (CEI UAM+CSIC) Isaac Newton, 8, 28760 Tres Cantos, Madrid, Spain

**Keywords:** silicon, power factor, boron, doping concentration sweep, sputtering, codeposition

## Abstract

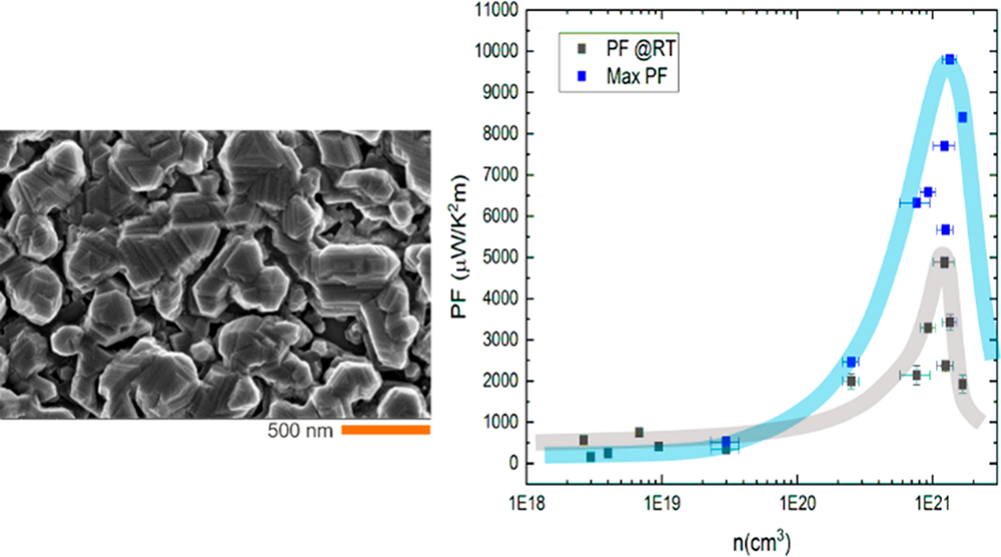

The power factor of highly boron-doped nanocrystalline Si thin films with controlled doping concentration is investigated. We achieve a high degree of tuning of boron content with a charge carrier concentration from 10^18^ to 10^21^/cm^3^ and with the electrical conductivity by varying the boron magnetron power from 10 to 60 W while maintaining the power of a SiB source constant during codeposition from two independent sputtering sources. Along with the increase in the electrical conductivity with increased boron doping, we observe a steady decrease in the Seebeck coefficient from 500 to 100 μV/K. These values result in power factors that exhibit a marked maximum of 5 mW/K^2^m for a carrier concentration of around 10^21^*/*cm^3^ at room temperature. Temperature-dependent measurements up to 650 °C show, with increasing doping concentration, a change of the resistivity from a semiconducting to a metallic behavior and an increase of both Seebeck coefficient and power factor, with this last one peaking at 9.8 mW/K^2^m in the 350–550 °C temperature range. For higher concentrations, scanning electron microscopy and energy-dispersive X-ray spectroscopy show a partial segregation of boron on particles on the surface. These results exemplify the great advantage of sputtering codeposition methods to easily tune and optimize the thermoelectric performance in thin films, obtaining in our specific case highly competitive power factors in a simple and reliable manner.

## Introduction

Thermoelectricity plays a substantial role in helping to solve the structural energy crisis in our society. The conversion to electricity of the residual heat waste generated by our machines and technology has a large potential for increasing the overall energy efficiency of the economy. In addition, thermoelectricity can be used to generate electricity ubiquitously, even from diffuse energy sources which are under-represented in our current energy mix, due to the modest generated temperature differences and low energy density.

Materialwise, Bi_2_Te_3_,^1,2^ PbTe,^3,4^ and SiGe^5−7^ can be considered the classical functional ingredients to constitute a thermoelectric device. The last two have dominated the application area representing the biggest success of thermoelectricity, space exploration, due to its use in radioisotope thermoelectric generators (RTG).^8−10^ Not being restricted to the three aforementioned compounds, material research has broadly expanded toward selenides,^11−14^ skutterudites,^15^ half-Heuslers-like (Hf,Ti,Zr)CoSb,^16^ or TiNiSn^17^ and full Heuslers, including the current development of Fe_2_VAl-related alloys.^18,19^

Some limiting aspects of this diverse family of thermoelectric materials related to the critical scarcity of some elements, environmental concerns over mining, and possible artificially generated bottlenecks due to geopolitical movements and national monopolies make the focus turn back to widely available materials such as the mentioned SiGe or even plain Si.^20−22^ For this last case, Narducci et al. recently reported on boron hyper-doped Si thin films with exceptional power factors (PF).^23^ Their obtained values are a result of the presence of carrier energy filtering in nanocrystalline Si thin films with a granular structure and intergrain barriers.^24−26^ Crystallinity here becomes another crucial factor as, for example, finely tuning crystallinity and doping of hydrogenated microcrystalline silicon thin films is also a keypoint into obtaining high-quality devices for above integrated circuit (above-IC) image sensors. From the thermoelectricity standpoint, although single-crystalline Si can also show high PF, it has a much larger thermal conductivity, which diminishes the thermoelectric performance,^27^ and therefore, nanocrystalline materials are obviously of great interest for high-output thermoelectrics. All this said, since the power factor is the result of the product σ·*S*^2^ and both quantities depend oppositely on doping concentration, a smart strategy to handle these opposite dependencies, combined with the adequate crystallinity, is required to maximize this parameter.

With this in mind, we used a physical vapor deposition technique that involved finely controlled boron enrichment of SiB films by codepositing boron and SiB from independent sputtering sources. In this way, Si thin films with varying boron content can easily be obtained. Besides, sputtering is a cheap, versatile, and industry-friendly technique, and its use, therefore, makes any future application of the present results for mass production realistic. In this work, the fabrication and characterization of the highly doped Si films are described, as well as their suitability for thermoelectrics.

We prove that the codeposition configuration enables good control of the boron content and charge carrier concentration. We show how this adjustment possibility allows for an optimization of the films in terms of the maximization of the PF values. Boron segregation effects are also studied with scanning electron microscopy (SEM) imaging and element-sensitive spectroscopy on the surface.

## Experimental Details

Polycrystalline boron-doped Si thin films with a thickness of 115 nm were deposited on Z-cut quartz substrates at 850 °C in a UHV chamber with a base pressure of 10^–10^ mbar. A codeposition configuration with two magnetron sputtering sources was used to tune the boron doping concentration in the films. DC magnetron sputtering was used for a boron-doped Si (SiB hereafter) target with a nominal electrical resistivity of 0.02–0.005 Ω·cm. This initial doping concentration was chosen to be the lowest in the range of interest, and for further and controlled boron enrichment, RF sputtering was used for the corresponding pure boron target. While the power of the SiB source was 100 W for all films, the boron source power was varied for different films from 0 W (no additional boron) to 60 W. Before this codeposition, a 10 nm thick Au prelayer was deposited by DC magnetron sputtering. Au was deposited at 850 °C, the same temperature as the SiB, to optimize the process time. The presence of this layer induces crystallization of the Si film, and the continuous migration of Au toward the surface during growth, owing to the high temperature values employed here, allows its easy removal by chemical means after deposition for electrical characterization.^6,28^ Ar pressure for the deposition of all the layers was 1.5 × 10^–3^ mbar.

The electrical conductivity and charge carrier concentration were measured with an HMS 5500 Hall effect measurement system (Ecopia). The temperature dependence of the electrical properties and thermoelectric parameters were studied with a Linseis LSR-3 Seebeck system. The crystalline properties were characterized by X-ray diffraction (XRD) with a Bruker D8 Discover X-ray system with a IμS source (Cu Kα1) and 2D detector. SEM imaging for surface characterization and energy-dispersive X-ray spectroscopy (EDX) were performed with SEM FEI Verios 460 equipment. A potassium iodide (KI) solution (Sigma Aldritch Au etchant 651842) was employed to remove Au after deposition. Samples were etched for 60 min and cleansed afterward with distilled water. The deposition temperature was determined by a conveniently located and calibrated thermocouple element in the commercial sample holder (UHV-Design).

## Film Growth and Crystalline Properties

To lower the high temperature required for the crystallization of Si,^28^ metal-induced crystallization (MIC) is used. Au has been successfully employed for MIC for Si^29^ and SiGe^5,6^ thin films, together with other metals such as Ag,^30^ Al,^31^ Ni,^32^ Cr,^33^ and Sn.^34^

The boron-doped Si films are grown onto the Au layer, which is dissolved into the growing film, forming a eutectic mixture. At the end of the process, the gold has completely migrated to the surface of the sample and can be removed with a KI etchant solution. This process is also referred to as “gold-induced layer exchange”^35,36^ or metal-induced crystallization. For this to occur, the sample temperature must be above the eutectic temperature for the Si–Au solution, which is around 360 °C.^37,38^ This MIC procedure with deposition at a controlled high temperature above the eutectic point is more advantageous than processes based on low-temperature deposition followed by annealing. This is justified by previous works on Si (not shown here) and on SiGe^6^ in which Au stays embedded in the film after annealing and cannot be removed, degrading the electric and thermoelectric properties.

**Figure 1:**
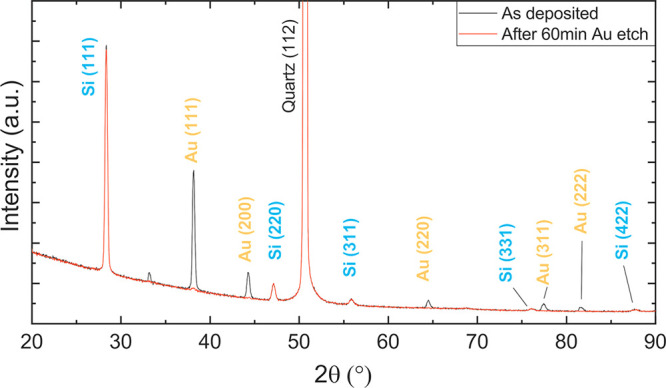
X-ray diffraction patterns of a 115 nm thick boron-doped Si film deposited with a boron RF power of 30 W using metal-induced crystallization with a Au layer. The black lines correspond to the as-deposited state, and the red line shows the situation after the chemical etching of the Au layer.

Figure 1 depicts a θ/2θ scan for a boron-doped Si film with an RF power of 30 W for the boron source in codeposition configuration. The graph shows the measurement for the as-deposited sample (black) and the same sample after the chemical etching of the Au layer with the KI solution (red). The characteristic Si(111) reflection is seen, together with (220), (311), and (422) reflections, proving the crystallization of the film in a polycrystalline phase. A crystallite size of 30 nm is obtained with the Scherrer equation. The data also show a polycrystalline nature of the Au layer. A preferential (111) orientation is assessed for both materials. After using the KI solution, almost all of the Au signal goes away, but the Si peaks do not change. In addition, electron backscattering imaging with a scanning electron microscope (EBS-SEM) on previously deposited samples at different temperatures (see Figure S1 in the Supporting Information) shows that for temperatures above 800 °C most of the Au particles trapped at lower temperatures have migrated to the surface and were removed by the KI solution. However, some unconnected particles stay embedded in the film. So we can conclude that, to the extent of this analysis, pure Au is efficiently eliminated from the silicon film at 850 °C.

## Electrical Properties

**Figure 2:**
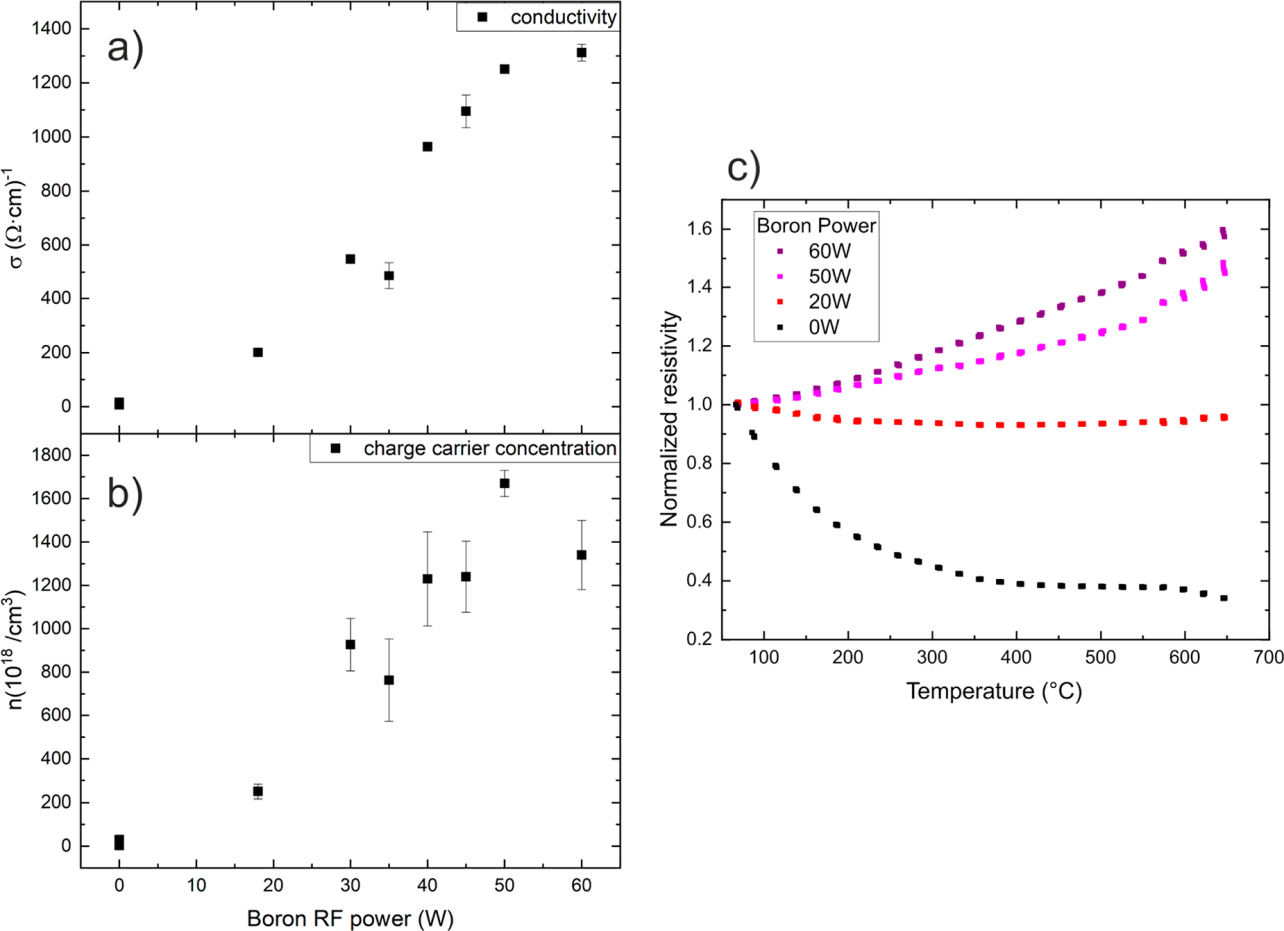
Dependence of the film conductivity (a) and the charge carrier concentration (b) on the RF power of the boron source. Data are obtained at room temperature. (c) Dependence of the normalized resistivity on the temperature for Si films with different boron content.

Figure 2a shows the dependence of the electrical conductivity at room temperature, measured using the Van der Pauw method, on the boron source power. The addition of boron results in a dramatic increase in the electrical conductivity by 2 orders of magnitude in the obtained films. This increase in conductivity with boron deposition power moderates at higher power values, nearly saturating at the highest powers. Figure 2b shows similar behavior for the charge carrier concentration at room temperature, confirming that the boron atoms are successfully incorporated in the Si films as charge carrier donors, and with a saturation effect at the highest boron RF powers. By changing the power of the boron source, the codeposition configuration makes it easy to control the amount of doping and charge carriers.

The temperature dependence of the resistivity for several films with different boron content is shown in Figure 2c. The value is normalized to the one at the lowest temperature value for visibility reasons due to the large changes shown in Figure 2a. For the 0 W case, i.e., with no additional boron enrichment to the film, the resistivity shows typical semiconducting behavior with a negative temperature coefficient. With increasing boron content, the temperature coefficient in the films flattens or even changes sign for the samples with larger boron content, showing a metal-like behavior. This shows that the intense doping concentration increase is strongly affecting the electronic structure of the material.

## Surface Morphology

**Figure 3:**
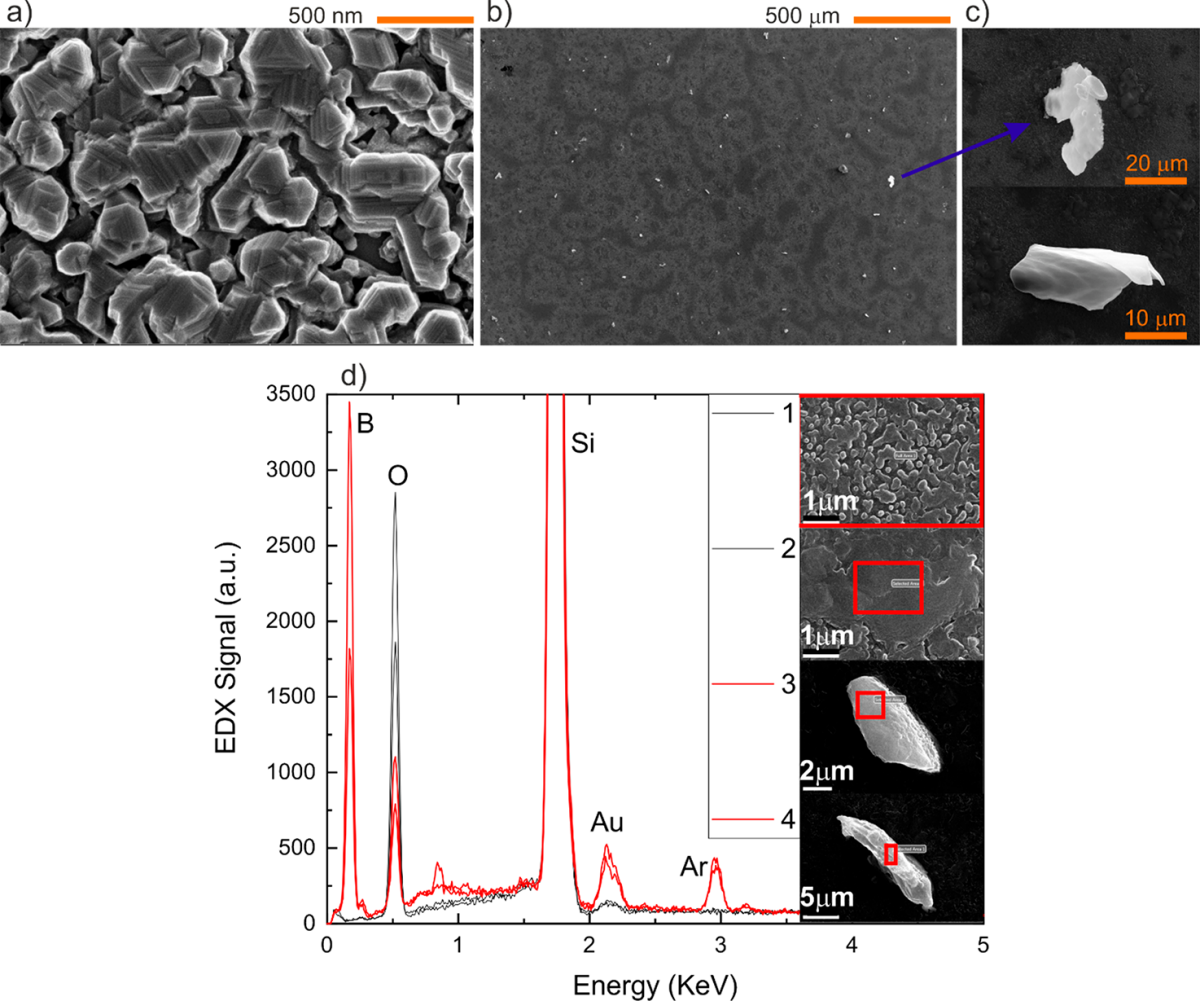
(a) SEM image showing the nanocrystalline nature of the Si thin films. The image corresponds to a sample with a boron RF power of 40 W. (b) Large-scale SEM image of a film deposited at 60 W, showing the formation of particles on the surface. (c) Detailed SEM images of the particles on the same sample. (d) EDX spectra for a sample deposited at 60 W boron source power with a high doping concentration. The image shows the spectra that were taken at four different areas of the sample. Spectra 1 and 2 (black) were measured in particle-free areas; 3 and 4 (red) were taken on the particles.

The surface morphology has been studied with SEM imaging. The morphology of the different films is the same, regardless of the boron content. Figure 3a shows an example image of the surface of one of the films in the series after Au removal deposited with a boron RF power of 40 W, with a granular structure formed by randomly oriented nanocrystallites, in agreement with the XRD data. The observed size of the crystallites also fits well with the value of 30 nm obtained with the Scherrer formula. Additional atomic force microscopy (AFM) imaging (see Supporting Information) show a root-mean-square roughness value of 60 nm. A 1000× zoomed-out image is shown in Figure 3b for a sample with a RF power of 60 W. At this scale, it is possible to see several particles on the surface with brighter contrast in the SEM image. These particles are also surrounded by slightly brighter areas, which overlap with each other. Detailed images of these particles from two different samples are shown in Figure 3c.

To gain insight into the nature and formation of these particles, EDX was employed to compare the composition of particle-free film regions and of the particles themselves. Figure 3d shows the EDX signal measured at four different positions on the surface of a Si film deposited with a boron RF power of 60 W. The images on the right show the measurement position and the red box represents the sampling area for the EDX signal (full area in case 1). In cases 1 and 2 (EDX curves in black), no signal from boron is found. However, when a zoom is made on a single particle, a distinctive and strong boron signal appears (EDX curves in red corresponding to cases 3 and 4). The absence of a boron signal in the plain film is not surprising, and it is owed to the very low sensitivity of EDX to boron and other light atoms. For the same reason, the large boron signal obtained on the particles is clear evidence of a large concentration of boron in them, much larger than the 2.2% atomic percentage corresponding to the nominal doping concentration. These data prove a process of partial segregation of the boron content to the surface and its accumulation in these particles as outgrowths. This explains the saturation effect observed in Figure 2a,b, where no additional charge carriers are created in spite of the larger relative amount of boron. This behavior was also confirmed in other samples with high boron doping.

Concerning the other peaks, a relatively larger Au signal is observed on the particles. This is probably the result of the inefficiency of the Au wet etching removal in or below the particles. The Ar signal is the result of the embedding of the atoms from the sputtering gas. The O and Si signals are both coming from the film and the quartz substrate, and therefore no additional conclusion can be drawn from them.

## Thermoelectric Performance

**Figure 4:**
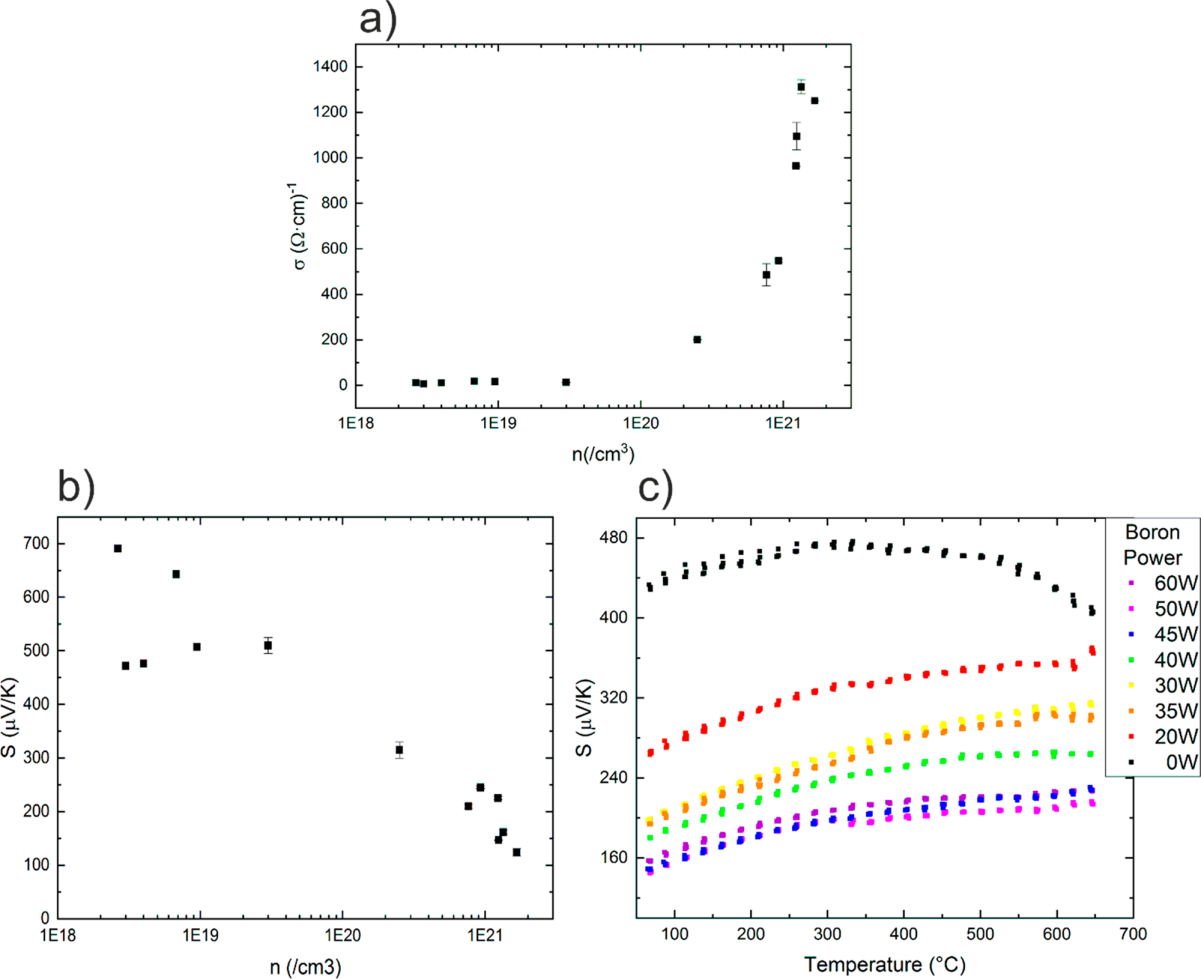
(a) Dependence of the Si thin film conductivity at room temperature on the charge carrier concentration. (b) Relationship of the measured Seebeck coefficient at room temperature with the charge carrier concentration. (c) Temperature dependence of the Seebeck coefficient for samples with varying boron content.

Figure 4a shows the dependence of the conductivity at room temperature on the charge carrier concentration in the deposited films. The qualitative behavior fits with the expected one from the theoretical models for semiconducting materials.^25,39−41^

The dependence of the Seebeck coefficient *S* at room temperature on the charge carrier concentration is shown in Figure 4b. It shows a steady decrease from values above 500 μV/K to values around 150 μV/K. This reduction in S is smaller in absolute value than the corresponding increase in the conductivity, but it has a large impact on the PF since it goes with a power of 2. Consequently, to maximize PF, a compromise is required. This is true for any temperature at which the thermoelectric material may be used. The temperature dependence of *S* was also measured, and it is shown in Figure 4c for different samples with different boron content labeled with the boron power source.

**Figure 5:**
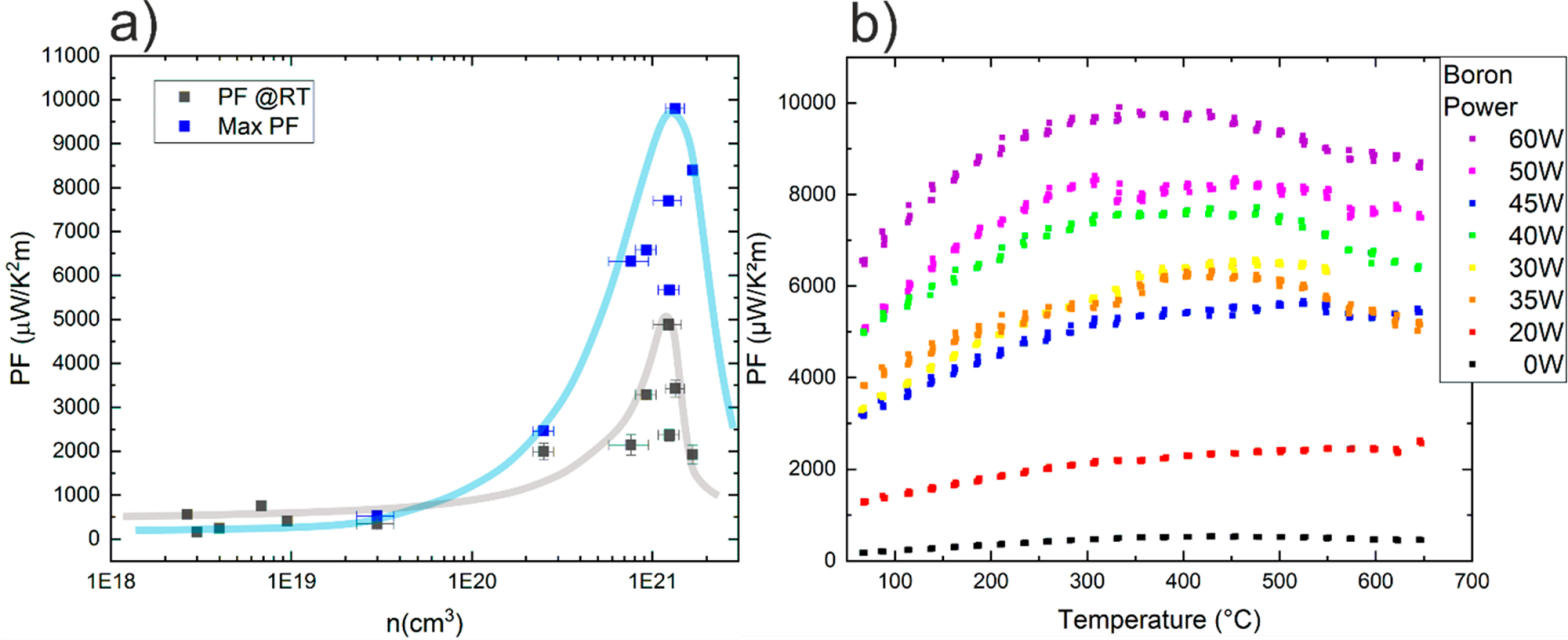
(a) Dependence of the PF on the charge carrier concentration. The lines are only a guide for the eye. (b) Dependence of the power factor PF on the temperature for samples with different boron content.

The dependence of the PF on the carrier concentration is plotted in Figure 5a. The lines are a guide for the eye. The values obtained at room temperature are shown in black; in blue, we show the largest obtained PF from its temperature dependence shown in Figure 5b. The dependence of PF on temperature shows a wide plateau around 350–550 °C, and the maximum values lay in this range.

Figure 5a shows that there is a narrow range of charge carrier concentration that maximizes the PF, in our case 1–1.1 × 10^21^/cm^3^. Narducci and Neophytou et al. showed that in Si nanocrystalline films with boron doping and, in general, granular overdoped semiconductors, the PF values are determined by an energy filtering effect and the localization of cold holes.^24−26^ For this, the formation of a potential barrier around the grains is needed. The grain size and shape and the height of the barrier strongly depend on the growth conditions and the segregation of the dopant to the intergrain area. This implies that the PF absolute values and the exact carrier concentration range where PF is maximized depend, not only on the material, but also on the exact deposition conditions and, eventually, on posterior annealing steps. For this reason, a cheap and versatile tool to vary the dopant concentration in a controlled manner is critical for the study and improvement of the thermoelectric performance of granular semiconductors. Since no relevant changes in grain size or morphology are observed for the different samples grown at different RF powers, we conclude that these parameters have no relevant direct effect in the increase on PF reported here. This study proves that sputtering in codeposition configuration fulfills these requirements, and it can be applied to almost any combination of semiconductor material and dopant atom. Also, since no hydrogen is involved in a sputter deposition process, there is no embedding of this element in the film, which has been shown to degrade the thermoelectric performance.^23^

In terms of PF absolute values, we obtain a maximum factor of 8.5–10 mW/K^2^m in the 350–550 °C temperature range and 4.9 mW/K^2^m at room temperature. These values are comparable to state-of-the-art (Bi,Pb)_2_Te, with PF spanning from 3 to 5 mW/K^2^m,^42−44^ showing the good thermoelectric performance of the films. On the other hand, to highlight the advantage of sputter codeposition as a fabrication route in a direct comparison, a PF of 1 mW/K^2^m was reported in boron-doped Si films deposited by chemical vapor deposition (CVD).^45^ Higher PF values of 16 mW/K^2^m for boron-doped Si films can be obtained after thermal treatment at 800–1000 °C for hydrogen removal and boron precipitation after CVD deposition.^23,46^ This thermal treatment approach has not been tested in our films, but it points to a strategy for further future improvement. This study also shows a large PF of 33 mW/K^2^m for samples annealed at 1000 °C after a previous 5 year aging step. Although this aging is by no means suitable for production purposes, it shows the huge potential of earth-abundant Si for thermoelectrics, and it is, to our knowledge, the largest reported PF for the material. In an industrial approach, nanocrystalline bulk samples prepared from compressed nanopellets were reported with PF values up to 2.5 mW/K^2^m.^47^ The PF values reported here are not only novel for boron-doped Si films deposited by sputtering, but they show the high potential of this technique, specifically in codeposition configuration, for the fabrication of thermoelectric materials. This potential includes the achievement of large carrier concentrations (≤10^21^*/*cm^3^). In fact, for Si, such values have been only reported for P-doped bulk samples made by spark plasma synthesis (SPS).^48^

Charge carrier concentration sweeps are scarce in the literature and are limited to a few materials. Ge doping was varied for Bi_2_O_2_Se (n-type), and opposite dependencies are reported for *S* and the electrical conductivity, as in our case, the PF increases to a maximum of 0.4 mW/K^2^m and decreases with larger doping.^49^ A maximum in the PF dependence is also reported for GeTe with Bi and Cu codoping, although this maximum is not present in all measured temperatures.^50^ In another telluride, PbTe, the maximum in PF is present for all temperatures in bulk samples.^51^ For GaN thin films, a maximum in PF is also observed although with a smaller value of 0.25 mW/K^2^m.^52^ An interesting study on boron- and phosphorus-doped Si thin films showed a similar dependence of the PF, but the fact that the sweep is limited to three samples prevents any deep comparison,^53^ and in any case, the maximum reported value for PF in that work for boron-doped Si is below 3 mW/K^2^m.

With one last important remark, a material with a charge carrier concentration optimized for maximum PF is not a material with a maximized figure of merit zT.^40,54^ The charge carrier concentration maximizing these two parameters is not generally the same. While the figure of merit zT rules the overall energy conversion efficiency, the PF rules the maximum electrical power generation. The latter is a more adequate parameter for applications with infinite heat sources, free heat sources (waste heat recovery), or high temperatures.^39,54−57^

## Conclusions

High-quality boron-doped Si thin films were grown in a codeposition sputtering configuration using metal-induced crystallization. The use of two independent material sources for SiB and boron allows for the controlled sweep of boron content and charge carrier concentration. Due to partial migration of boron to the surface eventually forming boron-rich aggregates, the carrier concentration and electrical conductivity can be changed at will until a saturation effect starts to happen. This is shown by SEM imaging and EDX spectroscopy.

The films have been optimized to maximize the power factor and the best performance is achieved for a carrier concentration of 1–1.1 × 10^21^/cm^3^, where PF values of 8.5–10 mW/K^2^m are obtained, resulting from a compromise between opposite trends in the dependence of the Seebeck coefficient and the electrical conductivity on the carrier concentration. The obtained PF values are a novelty for boron-doped Si films deposited by sputtering and are competitive with the ones obtained with other means. The high carrier concentration values above 10^21^/cm^3^ are achieved thanks to the use of the codeposition configuration and have not previously been reported for boron-doped Si films.

The versatility and industrial suitability of sputtering enable similar optimization queries in other materials and fast implementation in production processes.

## Supporting Information Available

Characterization of the Au presence in the films by EBS-SEM imaging of cross sections of the samples; additional sample characterization by Raman spectroscopy and surface characterization by AFM (PDF)
