# Supplementary material for: Sputtering Codeposition and Metal-Induced Crystallization to Enhance the Power Factor of Nanocrystalline Silicon
Source: ACS Appl Electron Mater. 2023 Apr 4;6(5):2799–806. doi: 10.1021/acsaelm.2c01772 (PMC11138147; doi:10.1021/acsaelm.2c01772)
Supplement: Supplementary file 1 — el2c01772_si_001.pdf [file el2c01772_si_001.pdf]

Sputtering co-deposition and metal-induced crystallization (MIC) to enhance the power factor of nanocrystalline silicon.

Andres Conca, Elías Ferreiro-Vila, Alfonso Cebollada, and Marisol Martin-Gonzalez*

Instituto de Micro y Nanotecnología, IMN-CNM, CSIC (CEI UAM+CSIC) Isaac Newton, 8, 28760, Tres Cantos, Madrid, Spain

E-mail: marisol.martin@csic.es

Before selecting the SiB growth temperature, a preliminary study on deposition temperature, from 400 to 850°C, was carried out. XRD data show that temperatures above 800ºC are required for good crystallization. This is demonstrated by the increased intensity and sharpness of the Si (111) reflection observed for higher temperatures, as well as the presence of the Si (311) and (220) peaks only at temperatures above 700°C.

The presence of Au in the film volume, which is not removable with the KI solution, has been studied in a deposition temperature series previous to the one presented in the main text. For this, electron backscattering imaging with a scanning electron microscope (EBSSEM) is used. The EBS-SEM images on cross-section of the samples is shown in Fig. S1 indicating the presence of embedded Au clusters in the film volume. These Au clusters increase the electrical conductivity while decrease the Seebeck coefficient and the power factor. For temperatures between 400-600ºC the Au is not migrating to the surface and short-circuits the film. At 700ºC the situation is improved but still a large Au content is observed. For 800ºC and above only single unconnected particles are seen. For temperatures above

800°C an adequate migration of the Au to the film surface is observed. The gold layer at the surface can then be removed well with KI solution.

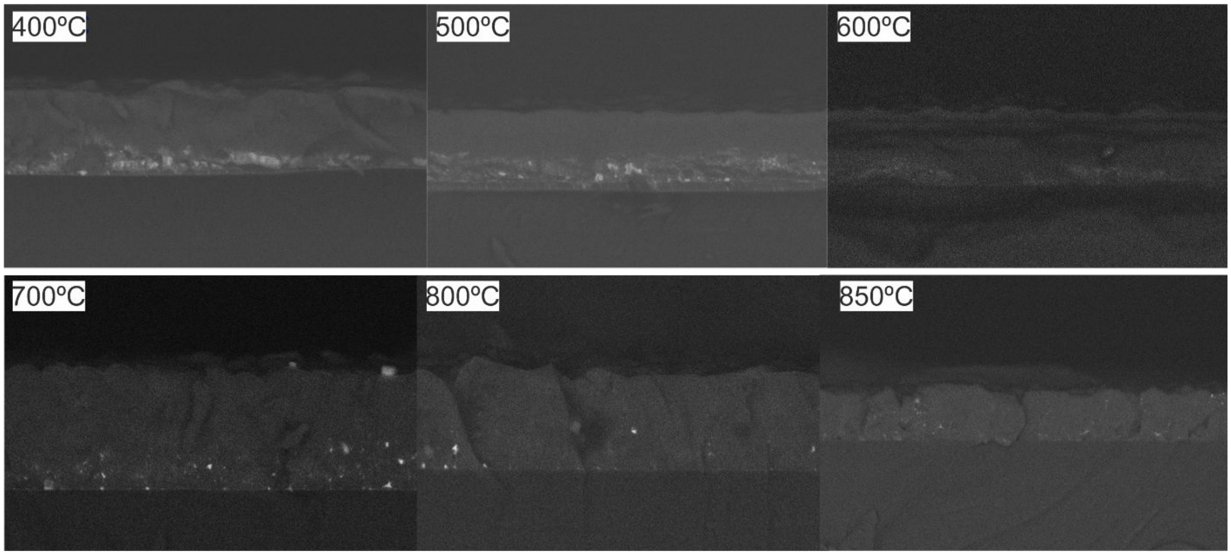

Figure S 1: Cross-section EBS-SEM of samples for different growth temperatures. The bright spots correspond to Au (due to the high atomic number) trapped inside the Si film.

In addition to the SEM imaging, the surface has been characterized with atomic force microscope (AFM). Two 5μm x 5μm images corresponding to two samples with different boron RF power are shown in Fig. S2. The observed granular structure is similar to the one seen in the SEM images. The RMS roughness of the samples is of 60nm.

Additional Raman data shown in Fig. S3 also support the use of deposition temperature above 800°C. The spectrum of a sample deposited at 600°C in Fig. S3a shows a very broad peak at 470cm^{-1} corresponding to amorphous Si compared to the sharp and high intense peaks at 530cm^{-1} for the 800°C and 700°C grown layers corresponding to crystallized Si. Moreover, Fig. S3b demonstrates the higher symmetry of the Si peak for the 800°C sample and the presence of a shoulder reminiscent of the amorphous Si for the 700°C sample. In conclusion, we can conclude from the XRD and Raman spectra measurements that the lower temperature samples (500°C and 600°C) present a low partial crystallization for the Si film and the fully crystallization of the Si film is obtained for the high temperature samples with an optimum crystallization at 800°C and above.

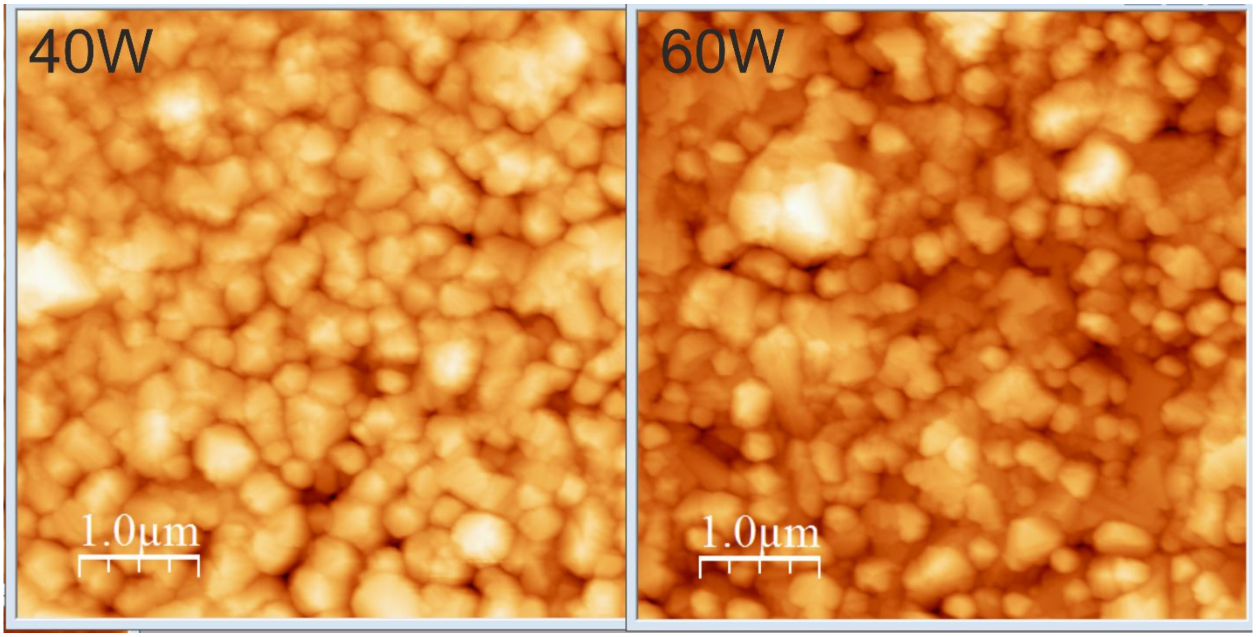

Figure S 2: AFM images (5μm x 5μm) of SiB films deposited with a RF power of 40 and 60W.

Fig. S4 shows, in double logarithmic scale, the dependence of the resistivity on the carrier concentration. The qualitative behavior and absolute values are similar to the ones reported for boron-doped Si.[1,2] The mobility values for the charge carrier in our samples are of 3-6 cm^2/Vs. These values do not change significantly with doping content. The values are lower than the ones reported for boron-doped Si,[2] owing probably to the granular structure of our films.

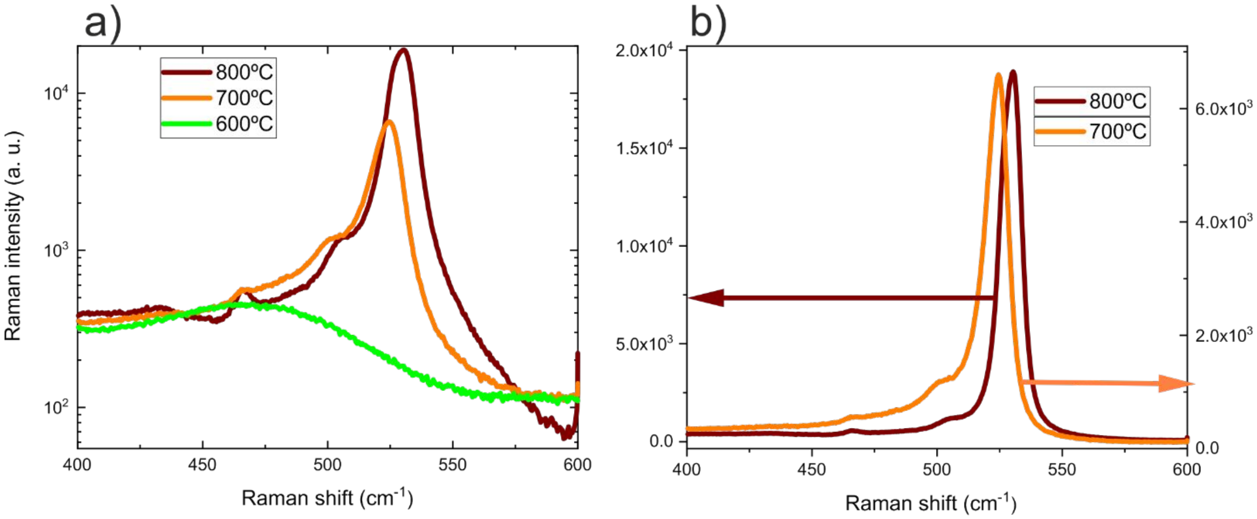

Figure S 3: a) Raman spectra for the 600°C, 700°C and 800°C samples. b) Linear scale for the highest temperature samples highlighting the asymmetry of the 700°C Si peak compared to the 800°C one.

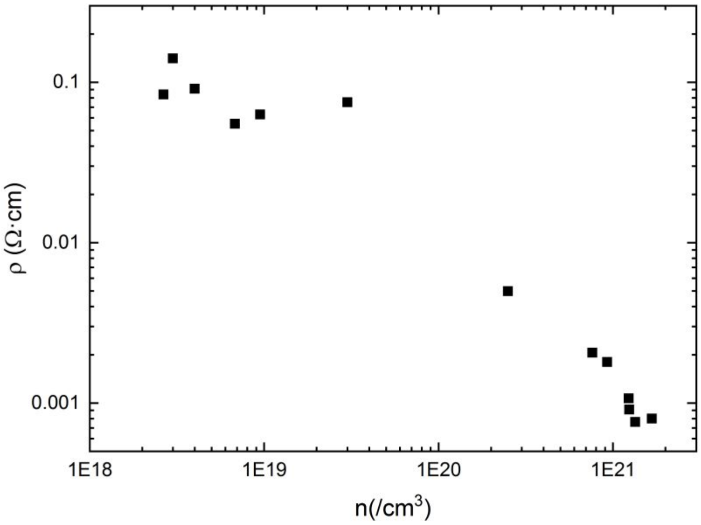

Figure S 4: Dependence of the resistivity of the samples on the carrier concentration.

References

(1) Thurber, W. R.; Mattis, R. L.; Liu, Y. M.; Filliben, J. J. Resistivity-Dopant Density Relationship for Boron-Doped Silicon. *Journal of The Electrochemical Society* **1980**, *127*, 2291.

(2) Thurber, W. R.; Mattis,; Liu,; Filliben, *The Relationship Between Resistivity and Dopant Density for Phosphorus- and Boron-Doped Silicon*; U.S. Department of Commerce National Bureau of Standards, 1981.
